# Exploring Prior Antibiotic Exposure Characteristics for COVID-19 Hospital Admission Patients: OpenSAFELY

**DOI:** 10.3390/antibiotics13060566

**Published:** 2024-06-18

**Authors:** Ya-Ting Yang, David Wong, Xiaomin Zhong, Ali Fahmi, Darren M. Ashcroft, Kieran Hand, Jon Massey, Brian Mackenna, Amir Mehrkar, Sebastian Bacon, Ben Goldacre, Victoria Palin, Tjeerd van Staa

**Affiliations:** 1 Centre for Health Informatics, School of Health Sciences, Faculty of Biology, Medicine and Health, The University of Manchester, Manchester M13 9PL, UK; 2 Leeds Institute of Health Sciences, The University of Leeds, Leeds LS2 9JT, UK; 3 Centre for Pharmacoepidemiology and Drug Safety, School of Health Sciences, Faculty of Biology, Medicine and Health, The University of Manchester, Manchester M13 9PL, UK; 4 National Institute for Health and Care Research (NIHR) Greater Manchester Patient Safety Translational Research Centre, School of Health Sciences, Faculty of Biology, Medicine and Health, The University of Manchester, Manchester M13 9PL, UK; 5 National Health Service (NHS) England, Wellington House, Waterloo Road, London SE1 8UG, UK; 6 Bennett Institute for Applied Data Science, Nuffield Department of Primary Care Health Sciences, University of Oxford, Oxford OX2 6GG, UK; 7 Division of Developmental Biology and Medicine, Maternal and Fetal Research Centre, St Marys Hospital, The University of Manchester, Manchester M13 9WL, UK

**Keywords:** antibiotics, COVID-19, primary care

## Abstract

Previous studies have demonstrated the association between antibiotic use and severe COVID-19 outcomes. This study aimed to explore detailed antibiotic exposure characteristics among COVID-19 patients. Using the OpenSAFELY platform, which integrates extensive health data and covers 40% of the population in England, the study analysed 3.16 million COVID-19 patients with at least two prior antibiotic prescriptions. These patients were compared to up to six matched controls without hospitalisation records. A machine learning model categorised patients into ten groups based on their antibiotic exposure history over the three years before their COVID-19 diagnosis. The study found that for COVID-19 patients, the total number of prior antibiotic prescriptions, diversity of antibiotic types, broad-spectrum antibiotic prescriptions, time between first and last antibiotics, and recent antibiotic use were associated with an increased risk of severe COVID-19 outcomes. Patients in the highest decile of antibiotic exposure had an adjusted odds ratio of 4.8 for severe outcomes compared to those in the lowest decile. These findings suggest a potential link between extensive antibiotic use and the risk of severe COVID-19. This highlights the need for more judicious antibiotic prescribing in primary care, primarily for patients with higher risks of infection-related complications, which may better offset the potential adverse effects of repeated antibiotic use.

## 1. Introduction

The COVID-19 pandemic has had an overwhelming impact worldwide. A meta-analysis reported that the global pooled case fatality rate was 1% among the general population in 2020 [1]. However, it is estimated that COVID-19 still caused 18.2 million excess deaths during the period from 2020 to 2021 [2]. 

According to Trougakos’s (2021) review, COVID-19 is a two-phase disease associated with mild or severe outcomes [3]. The first phase involves the virus spreading within the respiratory and gastrointestinal tracts, and the second phase may trigger a significant immune response in the hosts, where individual diversity (e.g., age and sex) drives the severity of the immune reaction. However, some potential factors related to severe outcomes may still be unknown. 

To date, several studies have observed altered gut microbiota accompanied by dysfunctional immune or metabolic responses related to severe COVID-19 infections in hospitalised patients [4,5,6]. Although there was no significant change in the gut microbiota for mild COVID-19 patients, virulence factors and antimicrobial resistance genes (ARGs) were found to have higher expression levels [7], which may lead to gut dysbiosis in these populations. 

Antibiotics are one of the major factors that can cause perturbation of the gut microbiome. A systematic review indicated that long-term dysbiosis may result from a combination of antibiotic types [8]. The administered antibiotic spectrum, route, and duration also influence the resilience of the gut microbiome [9]. Both our previous findings and research from Spain have shown frequent antibiotics might be correlated to COVID-19 severity [10], suggesting that antibiotic-associated alterations in the gut microbiota make the host susceptible to new infections. Although previous research analysed the volume and recent exposure timing of antibiotics, detailed prescribing patterns were not evaluated, which may help to understand how antibiotics impact the gut microbiota. This observed relationship may, in part, be confounded by a patient’s overall ill health, leading to a higher antibiotic prescribing history (e.g., in those who are immunosuppressed).

Therefore, the aim of this study was to build upon our previous research, which evaluated the effects of prior antibiotics on the severity of COVID-19 infection outcomes [11], to explore the relationship between COVID-19 infection severity and exposure to two or more prior antibiotic prescriptions. The primary goal was to more accurately describe the characteristics of antibiotic prescribing and its association with severe COVID-19 outcomes, taking into account various risk factors such as increased comorbidities in this population, age, sex, and ethnicity. 

## 2. Results

### 2.1. Study Participants

From 1 February 2020 to 31 December 2022, 3.2 million patients were identified as incident COVID-19 patients, and 889,850 (28.2%) patients had more than two antibiotic prescriptions in the 3-year exposure period. After matching, a balanced distribution in age, sex, region, and index date between case and control was achieved, resulting in 67,515 cases and 375,330 controls in the analysis (Supplementary Table S3). Table 1 shows that controls consisted of a healthier population at baseline. Cases had a higher proportion of unhealthy weight, presence of comorbidity, smoking history, and deprivation than controls. 

Supplementary Table S4 shows that cases had more antibiotic prescriptions (mean counts, 9.7 vs. 6.9), a longer antibiotic exposure period (mean days, 614.7 vs. 542.7), and more recent antibiotics received (mean days, 275.0 vs. 325.9). Over half of the control group received the lowest level of antibiotic types compared to cases (level 1, control 57.5% vs. case 49.5%), and most of the controls were not prescribed broad-spectrum antibiotics (level 1, control 73.9% vs. case 68.4%). Cases experienced shorter intervals between prescriptions, and controls showed less variability in prescribing intervals.

### 2.2. Antibiotic Exposure and Severe COVID-19 Outcome

**Figure 1:**
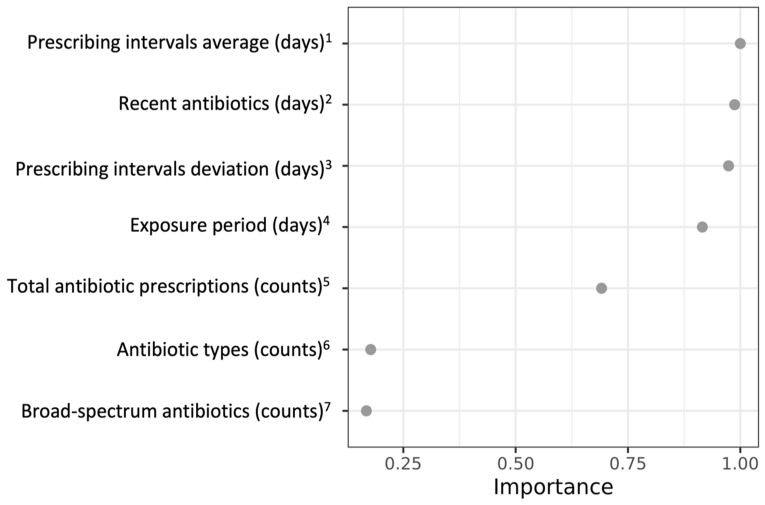
Variable importance plot based on the RF model. All antibiotic exposure variables were assessed in the prior 3 years, which started with 3 years plus 6 weeks before COVID-19 outcome and completed the whole 3-year observation. Antibiotics in the 6 weeks before the COVID-19 onset were excluded from the study design. Antibiotic-related variables included: ^1.^ The prescribing interval was estimated for each individual by collecting the number of days between each antibiotic prescription. The average of prescribing intervals was shown as the mean. ^2.^ time from the last antibiotic prescription until COVID-19 onset. ^3.^ The prescribing interval was estimated for each individual by collecting the number of days between each antibiotic prescription. The deviation of prescribing intervals was shown as the standard deviation. ^4.^ time between the first prescription and the last antibiotic prescription. ^5.^ count of the total number of antibiotic prescriptions. ^6.^ count of unique types of prior antibiotic prescriptions. ^7.^ count of prior broad-spectrum antibiotic prescriptions.

The prescribing interval average, recent antibiotic exposure, prescribing interval deviation, and exposure period were the most important variables in the random forest (RF) model (Figure 1). The RF model utilised antibiotic exposure data to stratify COVID-19 patients into decile groups based on their risk of hospitalisation. Higher decile groups, sharing similar antibiotic histories, have an increased risk of severe COVID-19 (Table 2). The adjusted conditional logistic regression (CLR) model revealed that those in the highest risk decile were approximately five times more likely to experience severe COVID-19 compared to those in the lowest risk decile. (adjusted model 4.8 [4.6–5.0]). 

**Figure 2:**
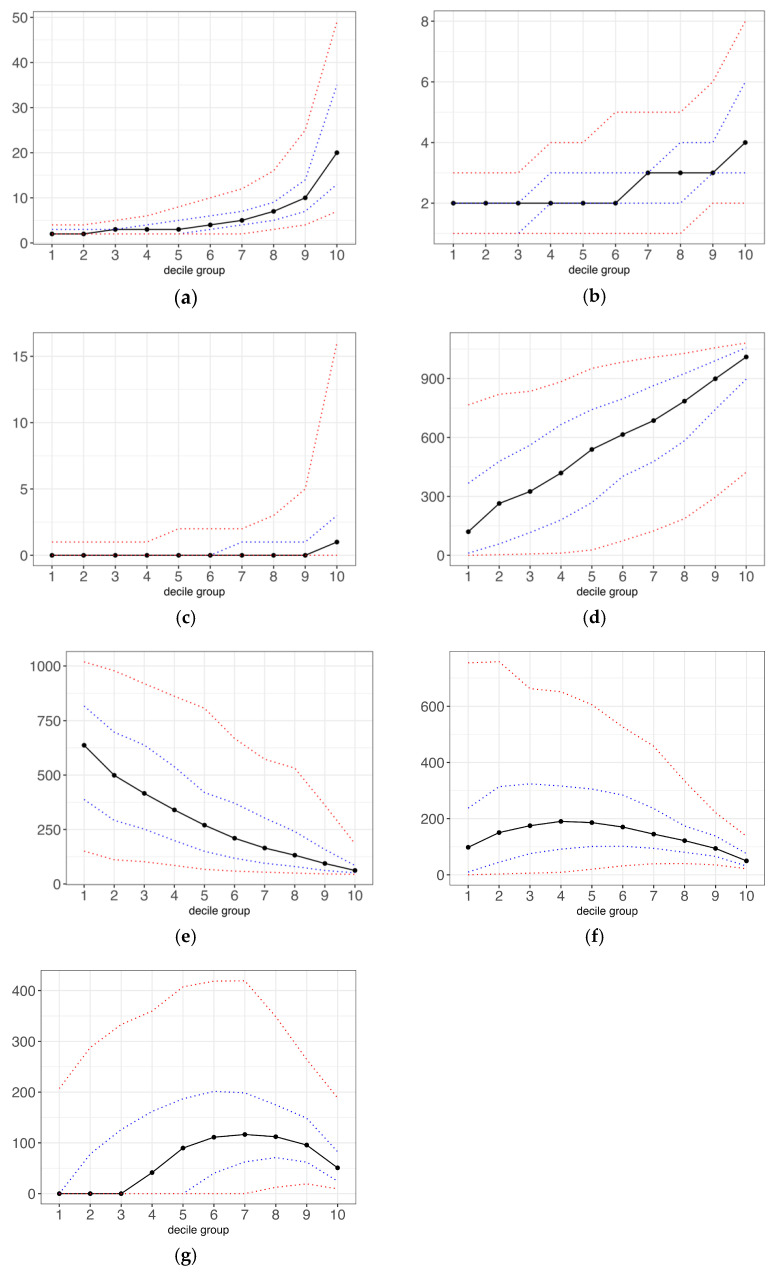
Distribution of antibiotic exposure characteristics across risk decile groups. (**a**) total antibiotics (counts); (**b**) antibiotic types (counts); (**c**) broad-spectrum antibiotics (counts); (**d**) time between (first to last antibiotics) (days); (**e**) recent antibiotics (days); (**f**) prescribing intervals average (days); (**g**) prescribing intervals deviation (days). All antibiotic exposure variables were assessed in the prior 3 years, which started from 3 years plus 6 weeks before the COVID-19 outcome and completed the whole 3-year observation. Antibiotics in the 6 weeks before COVID-19 onset were excluded from the study design. Antibiotic-related variables included: (**a**) count of total number of prior antibiotic prescriptions. (**b**) count of unique types of prior antibiotic prescriptions. (**c**) count of prior broad-spectrum antibiotic prescriptions. (**d**) time between the first prescription and the last antibiotic prescription. (**e**) time from the last antibiotic prescription until COVID-19 onset. (**f**,**g**) The prescribing interval was measured for each individual by collecting the number of days between each antibiotic prescription, and the mean and standard deviation were estimated. The black line showed the median value within each decile group; the blue line showed the 25th and 75th percentiles; and the red line showed the 5th and 95th percentiles.

Total antibiotics, antibiotic types, broad-spectrum antibiotics, and exposure period were found to increase with risk decile (Figure 2a–d), while recent antibiotics decreased (Figure 2e). Both the prescribing interval average and deviation presented rising and falling trends. However, the result shows that there was still a large amount of variability within each decile group. 

Table 3 summarises the combination of antibiotic characteristics by risk decile groups. Most patients in the lowest risk decile received the lowest level of total antibiotic prescriptions (73.1%), antibiotic types (91.9%), and broad-spectrum antibiotics (91.1%), and around 60% of patients had a shorter exposure period. On the other hand, patients in the highest risk decile received the most antibiotic prescriptions (92.8%), antibiotic types (66.5%), broad-spectrum antibiotics (41.9%), longest exposure period (79.5%), and most recent antibiotics (82.8%).

**Figure 3:**
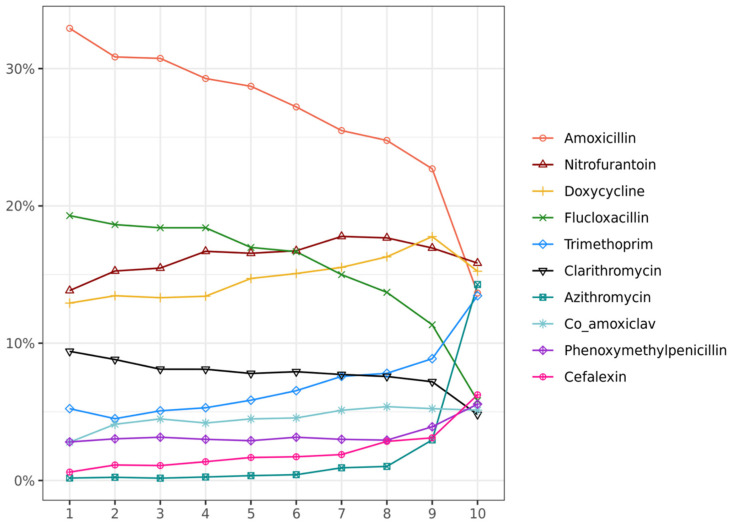
The proportion of antibiotic types in each decile group.

Figure 3 lists the 10 most common antibiotics: amoxicillin, nitrofurantoin, doxycycline, flucloxacillin, trimethoprim, clarithromycin, azithromycin, co-amoxiclav, phenoxymethylpenicillin, and cefalexin. The proportion of each type prescribed in each decile was measured. The consumption of different antibiotic types was closer in the highest decile group than in other deciles. Approximately 32.9% of the prescriptions prescribed in decile 1 were for Amoxicillin. Nitrofurantoin (13.8% to 16.4%) and Doxycycline (12.9% to 15.4%) were highly used and remained stable across the decile. It was noticeable that Azithromycin remained the lowest percentage from decile 1 to decile 9 (0.2% to 2.9%) but surged to 14.3% in decile 10. Trimethoprim presented an upward trend from 5.2% to 13.5% when decile increased.

### 2.3. Sensitivity Analysis for Antibiotic Exposure Interaction

In this sensitivity analysis, the interaction relationships between total antibiotic prescriptions and the other six antibiotic exposure variables were analysed (Supplementary Figure S2A–F). Generally, ORs increased with total antibiotic frequency subgroups from very low to very high users, but the trend varied within each subgroup. When compared to the lowest-level reference group, the ORs of each antibiotic type level were almost identical within subgroups. In contrast, the ORs increased with the levels of broad-spectrum antibiotics and exposure period and decreased with the levels of recent antibiotics within each subgroup. For average and deviation of prescribing intervals, ORs increased slightly in very low to high groups, while the highest ORs both appeared at level 1 in the very high subgroup and then went down with the levels. This suggested that for the most frequent antibiotic users, shorter days between repeated prescriptions were at higher risk.

## 3. Discussion

This study evaluated the association between the characteristics of antibiotics and the risk of hospital admission or death among patients with a recent COVID-19 infection who had received at least two antibiotic prescriptions in the prior three years. Variables such as the total number of antibiotic prescriptions, the number of antibiotic types, the number of broad-spectrum antibiotic prescriptions, the time between the first and last antibiotic prescriptions, as well as recent antibiotic use, were associated with becoming a case. The different characteristics of antibiotic exposure were found to be associated with the risk of severe COVID-19 outcomes. 

Compared to previous research in COVID-19 populations, the population in this study were patients who had used two or more antibiotics in the past three years. They exhibited a higher prevalence of white ethnicity, comorbidities, deprivation, residency in care homes, and a greater likelihood of receiving COVID-19 and influenza vaccinations. Nevertheless, after adjusting for confounding variables, these findings are consistent with previous research, which demonstrated that the quantity and diversity of antibiotic exposure were correlated with severe outcomes of COVID-19 infection [11]. This study also observed that patients at high risk of severe COVID-19 had received more recent antibiotics within the antibiotic assessment window. This variable, withdrawing prescriptions within the six weeks before COVID-19 onset, was implemented due to a meta-analysis’s conclusion that bacterial co-infections were less common in COVID-19 patients [12] and concerns regarding the overuse of antibiotics during the pandemic [13]. Considering that patients at higher risk of COVID-19 also had a longer duration between their first and last prescriptions, one plausible interpretation is that these high-risk patients were frequent and long-term users of antibiotics. However, patients with repeated antibiotic use tend to be more likely to be immunosuppressed and susceptible to infections, which may potentially confound the results. Furthermore, a review indicates that older age, males, and pre-existing comorbidities are linked to weaker immunity, which may contribute to the increased severity of COVID-19 [14]. Despite age and sex matching in this study, as well as adjustments made for comorbidities, it is essential to stay alert to potential factors that could influence the interpretation of the results.

A key question is what a possible biological mechanism could be behind an increased risk of viral infections (such as COVID-19) with larger and more diverse prior antibiotic exposures. Antibiotics can disrupt the microbiota in the gut as well as the oral and nasal cavities, resulting in a less diverse microbiota [8]. The imbalance of the intestinal microbiome may increase the susceptibility to infections by enabling opportunistic pathogens already present in the microbiota to thrive [15]. A systematic review by Elvers (2020) suggested that the acute alterations in the gut microbiome would recover to baseline within 1 month after ceasing commonly prescribed antibiotics in primary care, although other studies indicated a longer restitution time of 2–6 months [16]. The impact of repeated antibiotic exposures on the microbiota is less well studied. An animal model in rodents found that the gut microbiome disturbance lasted longer with a longer duration of exposure to amoxicillin [17]. Thus, intermittent and longer-duration antibiotic use may lead to frequent perturbations, affecting gut microbiota resilience. Furthermore, the gut microbiota serves as a reservoir for antibiotic resistance genes (ARGs), with prolonged treatment linked to increased antimicrobial resistance (AMR) risks [18,19]. The present study found that the level of broad-spectrum antibiotic prescribing was associated with a higher risk of severe COVID-19 outcomes. This is consistent with previous research that observed more multi-drug-resistant bacteria in a broad-spectrum antibiotic treatment group compared with the narrow-spectrum group [20]. Broad-spectrum antibiotics can also alter both bacterial and fungal compositions, which have complex interactions in maintaining the gut microbiome [21]. Consistent with our findings, several other observational studies have found associations between prior use of antibiotics and increased risks of infections and infection-related complications [10,11,22].

Another key question for assessing the harm-benefit ratio of repeated antibiotic exposure is whether frequent and diverse antibiotic exposure (with the highest risks in this study of severe COVID-19 outcomes) is actually effective in treating common bacterial infections and in reducing infection-related complications. Despite the widespread use of antibiotics and patients in primary care frequently getting multiple courses of an antibiotic over time [22], there is very limited substantive evidence from clinical trials to support this practice. For instance, about 29.1% of patients receive a repeat antibiotic prescription within 30 days of their initial prescription [23]. It has been reported that the bacteria of patients using an antibiotic are more likely to become resistant [24]. Despite this, treatment guidelines for common infections, such as those in England, seldom tackle the problems associated with frequent antibiotic prescribing, including diminishing effectiveness due to resistance and potential harm from adverse effects on the microbiota [25,26].

The strengths of this study include the use of OpenSAFELY, which covers over 40% of England’s population with comprehensive EHRs linked to COVID-19 data. Based on previous research that showed that patients with increased antibiotic usage were related to severe COVID-19 outcomes compared to patients without antibiotics [11], this study provides a more detailed description of antibiotic patterns related to severe COVID-19 by analysing the subgroups of COVID-19 patients with antibiotic use. Another advantage is the use of RF models, which are ideal for identifying high-risk patient subgroups and understanding exposure patterns. As this study attempts to address the combination of different antibiotic exposures, the complexities of regression models increase when considering interaction terms among all antibiotic-related variables. Instead, using Random Forest to create latent variables for summarisation is a more feasible approach.

This study also points out key limitations, including potential confounding factors and the inability to randomise participants’ antibiotic use, which might influence the study’s outcomes. First, the generalisation of this study might be limited to frequent antibiotic users rather than the general population (about 70% of the COVID-19 patients had only one or no antibiotic prescription). It was challenging to apply classification by including overall COVID-19 patients because redundant information from zero antibiotics predominated. In addition, antibiotic history is not a leading cause of severe COVID-19 but one of the risk factors that may deteriorate the outcome. Nevertheless, the observed harmful effects of repeated antibiotic use in this study should not be overlooked. Given that antibiotics and COVID-19 are both common in the community, this information will be beneficial for consideration in clinical decisions. Second, possible confounding due to reduced immune function in patients with a frequent history of antibiotic prescribing may have been present. However, the question is, even with the confounding present, whether frequent antibiotic use is actually beneficial in treating bacterial infections, considering it might lead to later adverse outcomes due to AMR or gut dysbiosis. Another consideration is that frequent antibiotic prescribing varies considerably between GPs, even after taking into account their case mix [27]. Finally, this study did not analyse the infection types, though the literature indicated that most of the antibiotics were poorly coded indications in primary care [27]. Future research could address this by exploring the interaction between infections and antibiotic prescribing, such as variability in antibiotic types or duration between antibiotic treatments.

In conclusion, the study found that in patients with antibiotic histories, a higher number of antibiotic prescriptions, a greater variety of antibiotic types, the use of broad-spectrum antibiotics, the duration between the first and last prescriptions, and recent antibiotic use are all factors that contribute to an increased risk of severe COVID-19. This highlights the need for more judicious antibiotic prescribing in primary care, primarily for patients with higher risks of infection-related complications, which may better offset the potential adverse effects of repeated antibiotic use. While the signals in this study of the potential harm of repeated antibiotic use are not proven causally, they do suggest repeated antibiotic use negatively influences one’s ability to fight future infections.

## 4. Materials and Methods

### 4.1. Data Sources 

On behalf of National Health Service (NHS), London, England, we used primary care records from OpenSAFELY-TPP, which comprises nearly 22 million patients’ electronic health records (EHRs), covering 40% of the population in England. Patient-level EHRs were linked to the Second Generation Surveillance System (SGSS), the NHS Digital Secondary Use Service (SUS), the COVID-19 Patient Notification System (CPNS), and the Office for National Statistics (ONS, London, UK) to acquire COVID-19 outcomes, including PCR tests, diagnosis of hospitalisation, and cause of death. Data linkage was provided via the OpenSAFELY integrated platform, which is governed by NHS England.

### 4.2. Study Design 

This was a matched case-control study. Eligible patients were newly identified as COVID-19 from SGSS positive tests, GP diagnosis records, or SUS hospital admission records from February 2020 to December 2022 (code lists are available in supplementary Table S1). Selection criteria included age from 18 to 110 years old, sex, registration with one GP practice for at least 3 years, and at least two antibiotic prescriptions within 3 years. The study design and the flowchart of patient selection are illustrated in Supplementary Figure S1. Patients who were admitted to the hospital for COVID-19 with a primary diagnosis code of U07.1 or U07.2 (International Classification of Diseases, 10th revision (ICD-10)) were categorised as a case group. Controls were defined as individuals without COVID-19-related hospital admissions (according to SUS data) or death records (from CPNS or ONS) within one month of their COVID-19 diagnosis.

### 4.3. Matching

Each case was matched with replacements for up to six eligible controls using the R package MatchIT v4.2.0 [28]. Matching variables included age (within a maximum range of 5 years), sex, region of GP practice, and index date (year and month). Age and sex partially accounted for individual susceptibility to COVID-19 and antibiotic exposure; region and index date accounted for the time and regional variation of COVID-19 infection and antibiotic prescribing.

### 4.4. Antibiotic Exposure

To measure the long-term impact of repeated antibiotic use on COVID-19 outcome severity, the maximum exposure time frame was set at three years, and antibiotic prescriptions in the most recent six weeks were not considered in the analysis because the acute effect of antibiotics was not a major concern in this study. Systemic antibiotics from the British National Formulary (BNF) chapter 5.1 (Antibacterial drugs), excluding both BNF 5.1.9 (Antituberculosis drugs) and BNF 5.1.10 (Antileprotic drugs), were included in the search strategy. There were 79 antibiotic compounds in the codelist, namely “antibiotic types”, and only 55 of them were identified in this study population. The code lists of all antibiotics and broad-spectrum antibiotics are listed in Supplementary Table S1.

Antibiotic exposure was summarised through the following variables: (i) the overall counts of antibiotic prescriptions; (ii) the number of unique types of antibiotics; (iii) the number of broad-spectrum antibiotics; (iv) the number of days between the first and last prescriptions; and (v) the time since the most recent antibiotic, measured as the number of days between the latest prescription and index date. The days between each antibiotic prescription during the exposure period were measured to capture the prescribing frequency, and the (vi) mean and (vii) standard deviation (SD) were calculated to indicate the average and variation of prescribing intervals. These continuous variables were categorised into four levels by quartile in descriptive statistics and sensitivity analysis. A detailed definition was included in Supplementary Table S2.

### 4.5. Confounding 

Comorbidities were considered important confounders since they were associated with severe COVID-19 complications and a possibly higher chance of receiving antibiotics. In this study, Charlson comorbidities were measured in the most recent 5 years, and the weighted score was divided into 5 subgroups (no comorbidities, low, medium, high, and very high) in the main analysis [29]. Other confounders recorded were ethnicity, body mass index (BMI), smoking status, care home residence, COVID-19, and influenza vaccination. All missing values were grouped as “unknown” as these clinical variables did not meet the “missing at random” assumption for imputation. 

### 4.6. Random Forest Model

Random Forest (RF) is a robust machine learning method for classification, relying on an ensemble of decision trees. It enhances accuracy through random sampling (bootstrapping) and feature selection for splits in each tree, making it effective for complex data analysis. Machine learning methods can identify clinical subgroups of patients with regard to outcomes [30] and can be used to classify risk levels of adverse outcomes [31]. Here, RF was applied to generate the probability of being a severe COVID-19 case using antibiotic exposure variables. The dataset was divided, with 80% for training and 20% for validation. After tuning and assessing the model, both sets were combined to refine the final model. The RF model hyperparameters included a 0.6 sample size, 3000 trees, a minimum node size of 800, and a depth of 500. Each split randomly picked 1 variable from a random set of 4 candidate variables by minimising Gini impurity, which enabled the maximisation of tree diversity and the minimisation of correlation between trees [32]. The contribution of each variable in the RF model was assessed by the mean decrease Gini score and presented as a variable importance plot. The visualisation ranked variables by their importance relative to the most important one, showcasing their descending order of influence. The RF model then calculated the probability of severe COVID-19 outcomes for individuals. Based on these probabilities, the study population was segmented into ten risk levels using decile cut-offs, with higher levels indicating a greater risk of severe outcomes. This approach allowed for a detailed examination of how antibiotic exposure varies across different risk levels.

### 4.7. Statistical Analysis 

Descriptive statistics described differences in baseline characteristics between case and control groups, as well as variations in antibiotic exposure patterns across decile groups. Patients in the same decile group exhibited similar antibiotic exposure, with higher deciles correlating to an increased risk of COVID-19 hospitalisation. The study further assessed the most common antibiotics across deciles. The association between risk deciles and COVID-19 hospitalisation was examined using conditional logistic regression (CLR) to calculate odds ratios (OR), with the first decile as the reference group. The model was adjusted for confounders including ethnicity, BMI, CCI, smoking, IMD, care home, COVID-19, and influenza vaccination. CLR was performed by R package survival v3.2-3, which considered matching strata for both the crude model and the model adjusted for confounders [33]. 

### 4.8. Sensitivity Analysis

The study analyses the total number of antibiotic prescriptions over three years as a key factor in understanding antibiotic exposure. This variable was crucial as it influenced other variables, such as the exposure period and prescribing interval deviation. To delve deeper, a sensitivity analysis was conducted to understand the interaction relationship between total antibiotics and other antibiotic exposure variables. Total prescriptions were grouped into quartiles, reflecting varying antibiotic use frequencies. CLR then assessed the odds ratio of hospitalisation risk across these subgroups.

### 4.9. Software and Reproducibility

If required, data management was performed using Python 3.9.1, with analysis carried out using R 4.0.5. Code for data management and analysis, as well as code lists, are archived online (https://github.com/opensafely/amr-uom-brit (accessed on 29 August 2023)). 

## Figures and Tables

**Table 1:** Baseline characteristics for cases and controls stratified by outcome.

	Case (n ^1^ = 67,515)		Control (n ^1^ = 375,330)	
	*n* ^1^	%	*n* ^1^	%
**Age group**				
18–29	1665	2.5	9115	2.4
30–39	3205	4.7	18,350	4.9
40–49	4735	7.0	27,040	7.2
50–59	8310	12.3	48,065	12.8
60–69	11,070	16.4	63,885	17.0
70–79	16,535	24.5	94,630	25.2
80+	21,995	32.6	114,245	30.4
**Sex**				
male	36,555	54.1	207,450	55.3
female	30,960	45.9	167,880	44.7
**Ethnicity**				
White	57,400	85.0	305,410	81.4
South Asian	5495	8.1	28,565	7.6
Black	1310	1.9	4135	1.1
Mixed	560	0.8	2605	0.7
Other	1145	1.7	4760	1.3
Unknown	1600	2.4	29,860	8.0
**BMI category ^2^**				
Healthy weight (<18.5 kg/m^2^)	13,490	20.0	82,845	22.1
Underweight (18.5–24.9 kg/m ^2^)	1580	2.3	7275	1.9
Overweight (25–29.9 kg/m ^2^)	16,935	25.1	109,010	29.0
Obese (≥30 kg/m^2^)	24,730	36.6	112,190	29.9
Unknown	10,780	16.0	64,010	17.1
**CCI group ^3^**				
No comorbidities (0)	19,830	29.4	164,015	43.7
Low (1–2)	36,765	54.5	177,195	47.2
Medium (3–4)	9685	14.3	30,845	8.2
High (5–6)	1185	1.8	3085	0.8
Very high (≥ 7)	50	0.1	195	0.1
**Smoking status ^4^**				
Never	22,425	33.2	142,935	38.1
Current	6145	9.1	34,385	9.2
Former	38,790	57.4	196,805	52.4
Unknown	160	0.2	1215	0.3
**IMD ^5^**				
1 (least deprived)	9370	13.9	62,170	16.6
2	11,470	17.0	70,280	18.7
3	13,420	19.9	77,720	20.7
4	14,380	21.3	77,020	20.5
5 (most deprived)	17,605	26.1	79,830	21.3
Unknown	1265	1.9	8305	2.2
**Care home residents**	3010	4.5	31,875	8.5
**COVID-19 vaccine ^6^**	29,100	43.1	181,090	48.2
**Flu vaccine ^7^**	46,285	68.6	261,090	69.6

^1.^ The counts of patients were rounded to the nearest five numbers in line with disclosure controls. ^2.^ BMI, or body mass index as most recent recorded within the previous 5 years. ^3.^ CCI, Charlson Comorbidities Index, measured from 17 weighted conditions, including Myocardial infarction, Congestive heart failure, Peripheral vascular disease, Cerebrovascular disease, Dementia, Chronic pulmonary disease, Connective tissue disease, Ulcer disease, Mild liver disease, Diabetes, Hemiplegia, Moderate or severe renal disease, Diabetes with complications, Any malignancy (including leukaemia and lymphoma), Moderate or severe liver disease, Metastatic solid tumour, and AIDS. ^4.^ Smoking status and care home residents identified from the most recent clinical records. ^5.^ IMD (Index of Multiple Deprivation) quintile measured from patient-level postcode. ^6.^ COVID-19 vaccine has been identified since the vaccination programme started. ^7.^ Influenza vaccine identified in the previous 2 years.

**Table 2:** Association of prior antibiotic exposure levels and COVID-19 outcomes.

	Probability of COVID-19 Hospitalisation		Conditional Logistic Regression Model ^1^	
Risk Level	RF Estimated	Observed	OR	95% CI
Decile 1 (lowest)	0.09	0.08	ref	
Decile 2	0.11	0.10	1.3	1.2–1.3
Decile 3	0.12	0.11	1.5	1.4–1.5
Decile 4	0.13	0.12	1.6	1.5–1.7
Decile 5	0.14	0.13	1.7	1.6–1.8
Decile 6	0.15	0.14	1.9	1.8–2.0
Decile 7	0.16	0.16	2.1	2.1–2.2
Decile 8	0.17	0.18	2.6	2.5–2.7
Decile 9	0.19	0.22	3.1	3.0–3.2
Decile 10 (highest)	0.25	0.30	4.8	4.6–5.0

^1.^ adjusted for ethnicity, BMI category, CCI group, smoking status, IMD, care home residents, COVID-19, and influenza vaccine.

**Table 3:** Antibiotic characteristics by RF decile groups.

Variables ^1,2,3^	Decile 1 (Lowest Risk)	Decile 2	Decile 3	Decile 4	Decile 5	Decile 6	Decile 7	Decile 8	Decile 9	Decile 10 (Highest Risk)
**Total antibiotics (count) ^4^**	2 (2, 3)	2 (2, 3)	3 (2, 3)	3 (2, 4)	3 (2, 5)	4 (3, 6)	5 (4, 7)	7 (5, 9)	10 (7, 14)	20 (13, 35)
Level 1 (2) (lowest)	73.1%	58.5%	48.8%	38.8%	26.4%	15.1%	7.6%	2.1%	0.2%	0%
Level 2 (3)	25.6%	37.7%	43.3%	45.9%	45.3%	41.4%	30.3%	16.6%	5.6%	0.6%
Level 3 (6)	1.3%	3.5%	7.2%	13%	22.3%	32%	39.5%	38.6%	68.5%	6.6%
Level 4 (13) (highest)	0%	0.3%	0.6%	2.3%	6%	11.5%	22.6%	42.7%	23.7%	92.8%
**Antibiotic types (count) ^5^**	2 (1, 2)	2 (1, 2)	2 (1, 2)	2 (2, 3)	2 (2, 3)	2 (2, 3)	2 (2, 3)	3 (2, 4)	3 (3, 4)	4 (3, 6)
Level 1 (2) (lowest)	91.9%	83.9%	78.7%	73.6%	64.1%	53.8%	44.9%	33.7%	23.7%	14.8%
Level 2 (3)	7.2%	14.1%	17.2%	19.3%	23.4%	28.8%	31.5%	31.6%	27.3%	18.7%
Level 3 (4) (highest)	0.8%	2.0%	4.2%	7.1%	12.5%	17.4%	23.6%	34.8%	49.0%	66.5%
**Broad-spectrum antibiotics (count) ^6^**	0 (0, 0)	0 (0, 0)	0 (0, 0)	0 (0, 0)	0 (0, 0)	0 (0, 0)	0 (0, 1)	0 (0, 1)	0 (0, 1)	1 (0, 3)
Level 1 (0) (lowest)	91.1%	85.3%	83.5%	82.6%	80.6%	77.0%	71.5%	64.0%	55.9%	39.3%
Level 2 (1)	7.2%	12.1%	13.3%	13.6%	14.3%	15.8%	17.3%	19.9%	21.4%	18.8%
Level 3 (3) (highest)	1.7%	2.6%	3.2%	3.8%	5.1%	7.2%	11.3%	16.1%	22.7%	41.9%
**Time between (day) ^7^**	120 (11, 367)	264 (58, 478)	325 (116, 561)	419 (180, 666)	539 (268, 472)	615 (402, 797)	686 (477, 864)	785 (583, 925)	899 (743, 991)	1010 (897, 1057)
Level 1 (75) (lowest)	63.1%	48.9%	42.0%	32.9%	23.9%	15.4%	10.3%	7.3%	4.0%	2.1%
Level 2 (423)	24.9%	33.2%	35.1%	34.9%	31.8%	30.7%	26.3%	17.9%	9.3%	6.2%
Level 3 (728)	10.0%	14.5%	18.5%	25.9%	32.5%	36.4%	37.0%	36.6%	26.9%	12.1%
Level 4 (977) (highest)	2.0%	3.4%	4.4%	6.3%	11.8%	17.4%	26.3%	38.2%	59.8%	79.5%
**Recent antibiotics (day) ^8^**	637 (388, 817)	499 (292, 697)	416 (252, 638)	340 (199, 538)	270 (150, 420)	210 (188, 371)	165 (95, 303)	132 (80, 241)	94 (62, 158)	62 (51, 85)
Level 1 (65) (lowest)	2.5%	3.6%	4.8%	7.4%	13.1%	20.0%	27.4%	36.0%	53.5%	82.8%
Level 2 (155)	6.8%	12.9%	17.5%	24.0%	30.3%	33.9%	37.1%	38.0%	33.6%	14.8%
Level 3 (334)	23.6%	30.0%	34.4%	37.0%	36.8%	32.7%	26.0%	18.5%	9.8%	1.9%
Level 4 (678) (highest)	67.1%	53.5%	43.3%	31.6%	19.8%	13.4%	9.6%	7.5%	3.0%	0.5%
**Prescribing intervals average (day) ^9^**	98 (10, 238)	151 (45, 314)	175 (76, 324)	190 (92, 316)	186 (101, 306)	170 (102, 284)	145 (95, 236)	122 (80, 174)	94 (66, 138)	50 (31, 76)
Level 1 (30) (lowest)	43.7%	29.8%	22.2%	17.2%	13.3%	11.6%	11.8%	14.6%	22.0%	63.8%
Level 2 (93)	13.5%	15.3%	16.6%	17.1%	19.1%	23.4%	29.8%	38.4%	47.2%	29.6%
Level 3 (171)	17.6%	20.6%	24.2%	26.3%	29.2%	31.3%	33.5%	34.1%	27.1%	6.0%
Level 4 (363) (highest)	25.1%	34.3%	37.0%	39.4%	38.5%	33.7%	24.9%	12.8%	3.7%	0.6%
**Prescribing intervals deviation (day) ^9^**	0 (0, 0)	0 (0, 78)	0 (0, 126)	41 (0, 162)	90 (0, 187)	111 (40, 202)	117 (63, 199)	112 (71, 175)	96 (62, 149)	51 (25, 83)
Level 1 (0) (lowest)	77.0%	62.1%	52.2%	41.2%	27.8%	16.1%	8.6%	2.9%	0.7%	0.2%
Level 2 (37)	7.6%	11.7%	12.8%	14.8%	16.3%	18.0%	18.2%	20.0%	27.9%	64.1%
Level 3 (103)	6.0%	10.7%	14.0%	16.8%	22.4%	28.3%	35.0%	43.0%	46.2%	27.6%
Level 4 (226) (highest)	9.4%	15.5%	21.0%	27.2%	33.5%	37.6%	38.2%	34.2%	25.3%	8.2%

^1.^ continuous variables: mean (SD); and grouped by levels as categorical variables: patient number and percentage. ^2.^ The values of each decile were shown as the median and Q1 (25th percentile) and Q3 (75th percentile) numbers; the values of each antibiotic exposure level 1–4 show the median value in brackets. ^3.^ The counts of patients were rounded to the nearest five numbers in line with disclosure controls. ^4.^ count of total antibiotic prescriptions grouped by quartile; level 1 is the lowest (1st quartile), and level 4 is the highest quartile (4th quartile). ^5.^ count of unique antibiotic types grouped by quartile; level 1 is the lowest (combined 1st and 2nd quartile for the same value); level 3 is the highest quartile (4th quartile). ^6.^ count of broad-spectrum antibiotic prescriptions grouped by quartile; level 1 is the lowest (combined 1st and 2nd quartile for the same value); level 3 is the highest quartile (4th quartile). ^7.^ The time between the first prescription and the last antibiotic prescription was estimated and grouped by quartile; level 1 is the lowest (1st quartile), and level 4 is the highest quartile (4th quartile). ^8.^ From the last antibiotic prescription until COVID-19 onset, days were estimated and grouped by quartile; level 1 is the lowest (1st quartile), and level 4 is the highest quartile (4th quartile). ^9.^ The prescribing interval was measured by collecting the number of days between each antibiotic prescription by individuals, and then the mean and standard deviation were estimated. The values were grouped by quartile; level 1 is the lowest (1st quartile), and level 4 is the highest quartile (4th quartile).

## Data Availability

Access to the underlying identifiable and potentially re-identifiable pseudonymised electronic health record data is tightly governed by various legislative and regulatory frameworks and restricted by best practices. The data in OpenSAFELY is drawn from General Practice data across England, where TPP is the Data Processor. TPP developers (CB, JP, FH, SH, JC) initiate an automated process to create pseudonymised records in the core OpenSAFELY database, which are copies of key structured data tables in the identifiable records. These are linked to key external data resources that have also been pseudonymised via SHA-512 one-way hashing of NHS numbers using a shared salt. DataLab developers and PIs holding contracts with NHS England have access to the OpenSAFELY pseudonymised data tables as needed to develop the OpenSAFELY tools. These tools in turn enable researchers with OpenSAFELY Data Access Agreements to write and execute code for data management and data analysis without direct access to the underlying raw pseudonymised patient data and to review the outputs of this code. All code for the full data management pipeline—from raw data to completed results for this analysis—and for the OpenSAFELY platform as a whole is available for review at github.com/OpenSAFELY. The data management and analysis code for this paper was led by YY and contributed by JM, VP, XZ, and AF. All data were linked, stored, and analysed securely within the OpenSAFELY platform https://opensafely.org/. Data include pseudonymised data such as coded diagnoses, medications, and physiological parameters. No free text data is included. All code is shared openly for review and re-use under the MIT open license (https://github.com/opensafely/amr-uom-brit/ (accessed on 4 July 2023)). Detailed pseudonymised patient data is potentially re-identifiable and therefore not shared. We rapidly delivered the OpenSAFELY data analysis platform without prior funding to deliver timely analyses on urgent research questions in the context of the global COVID-19 health emergency. Now that the platform is established, we are developing a formal process for external users to request access in collaboration with NHS England; details of this process will be published shortly on OpenSAFELY.org.

## Supplementary Materials

The following supporting information can be downloaded at: https://www.mdpi.com/article/10.3390/antibiotics13060566/s1. Supplementary Figure S1A. Diagram of patient selection (index date) and prior antibiotic measurements (AB exposure in 3 years); Supplementary Figure S1B. Flowchart of the patient selection process. Supplementary Figure S2A–F. Sensitivity analysis: interaction between total antibiotic prescriptions and other antibiotic exposure variables. Supplementary Table S1. Code lists are used for variable definition. Supplementary Table S2: Definition of antibiotic-related exposure variables. Supplementary Table S3. Characteristics of study cohorts before and after matching. Supplementary Table S4: Antibiotic exposure stratified by outcome.
