# Supplementary material for: Exploring Prior Antibiotic Exposure Characteristics for COVID-19 Hospital Admission Patients: OpenSAFELY
Source: Antibiotics (Basel). 2024 Jun 18;13(6):566. doi: 10.3390/antibiotics13060566 (PMC11201135; doi:10.3390/antibiotics13060566)
Supplement: Supplementary file 1 [file antibiotics-13-00566-s001.zip › antibiotics-3047369-supplementary.pdf]

Supplementary Contents

Figures *2*

Supplementary Figure S1A. Diagram of patient selection (index date) and prior antibiotic measurements (AB exposure in 3 years); Supplementary Figure S1B. Flowchart of patient selection process 2

Supplementary Figure S2A-F. Sensitivity analysis: interaction between total antibiotic prescriptions and other antibiotic exposure variables 3

Tables *6*

Supplementary Table S1. Code lists are used for variable definition. 6

Supplementary Table S2: Definition of antibiotic-related exposure variables 8

Supplementary Table S3. Characteristics of study cohorts before and after matching 9

Supplementary Table S4: Antibiotic exposure stratified by outcome 10

Figures

Supplementary Figure S1A. Diagram of patient selection (index date) and prior antibiotic measurements (AB exposure in 3 years); Supplementary Figure S1B. Flowchart of patient selection process

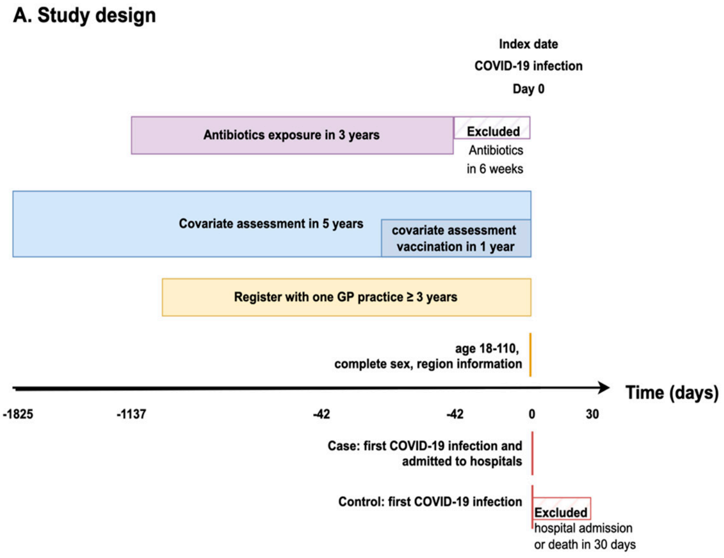

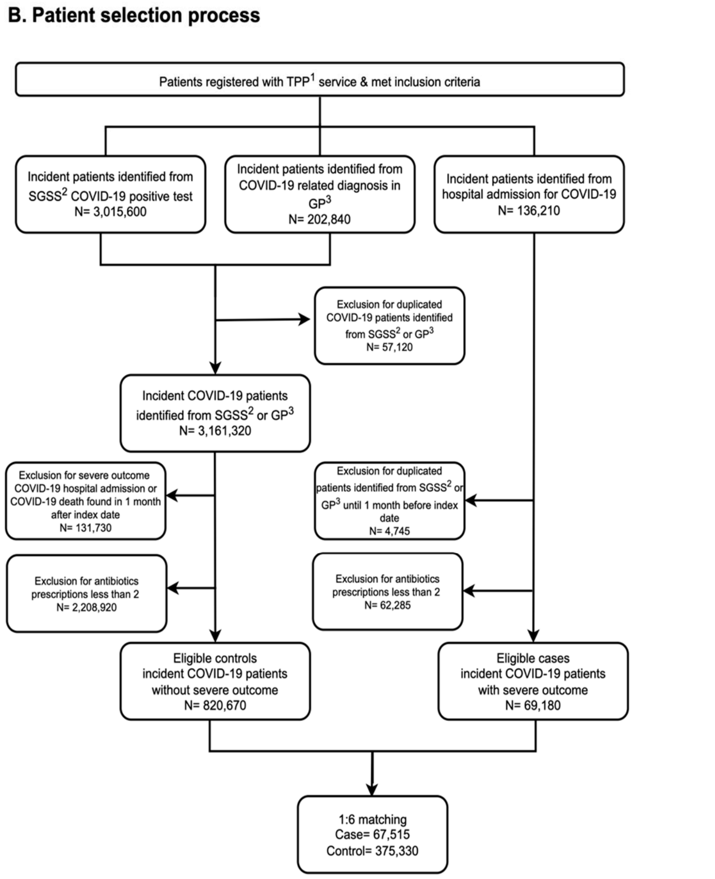

The counts of patients were round to nearest 5 number in line with disclosure controls.

1. TPP is an electronic health record system supplier; 2. SGSS, Second Generation Surveillance System;
3. GP, General Practice

Supplementary Figure S2A-F. Sensitivity analysis: interaction between total antibiotic prescriptions and other antibiotic exposure variables

A. Antibiotic types are stratified by the frequency of total antibiotics

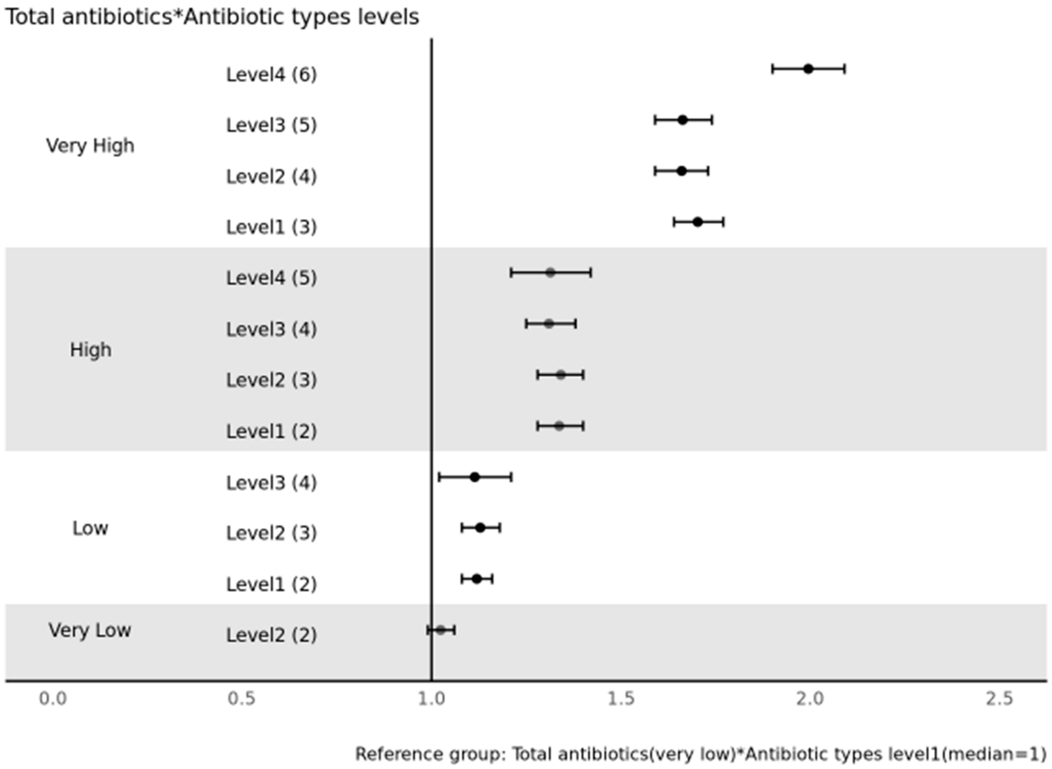

B. Broad-spectrum antibiotics stratified by frequency of total antibiotics

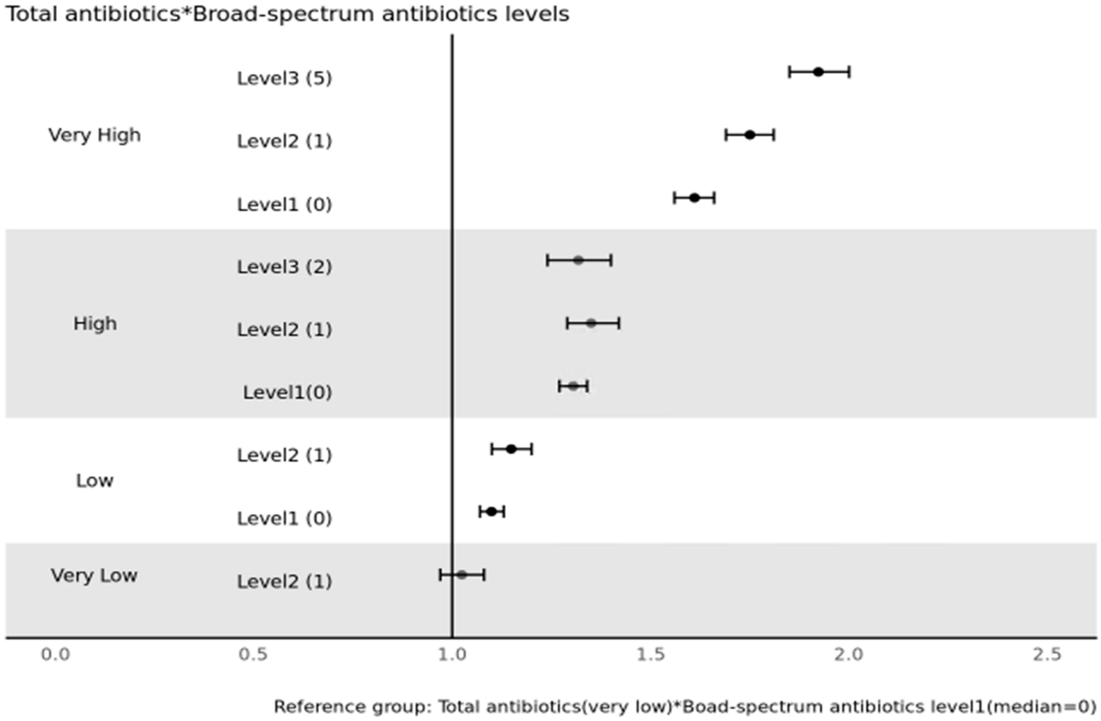

C. Exposure period stratified by frequency of total antibiotics

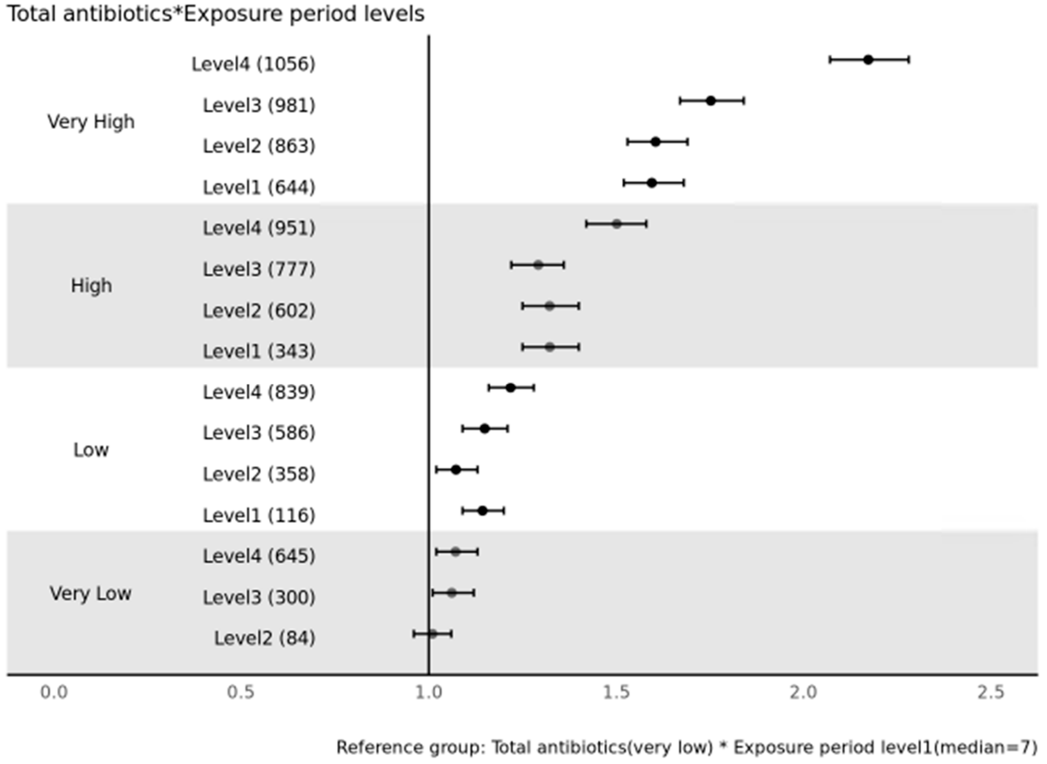

D. Recent antibiotics stratified by frequency of total antibiotics

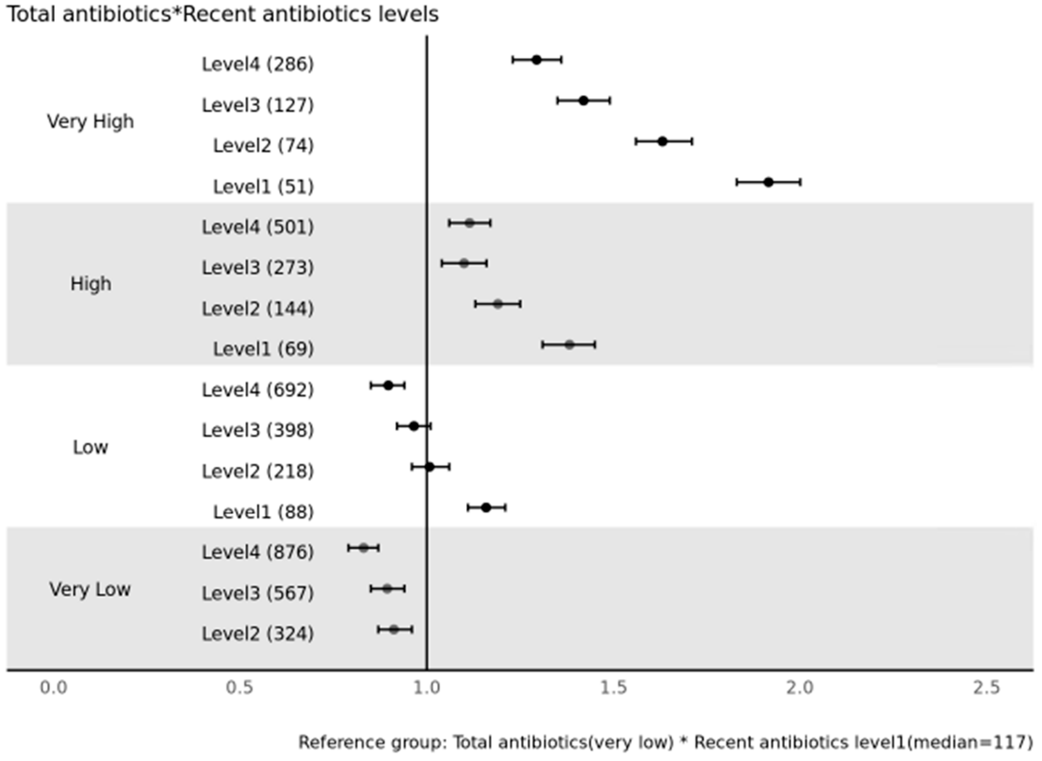

E. Prescribing intervals are average stratified by frequency of total antibiotics

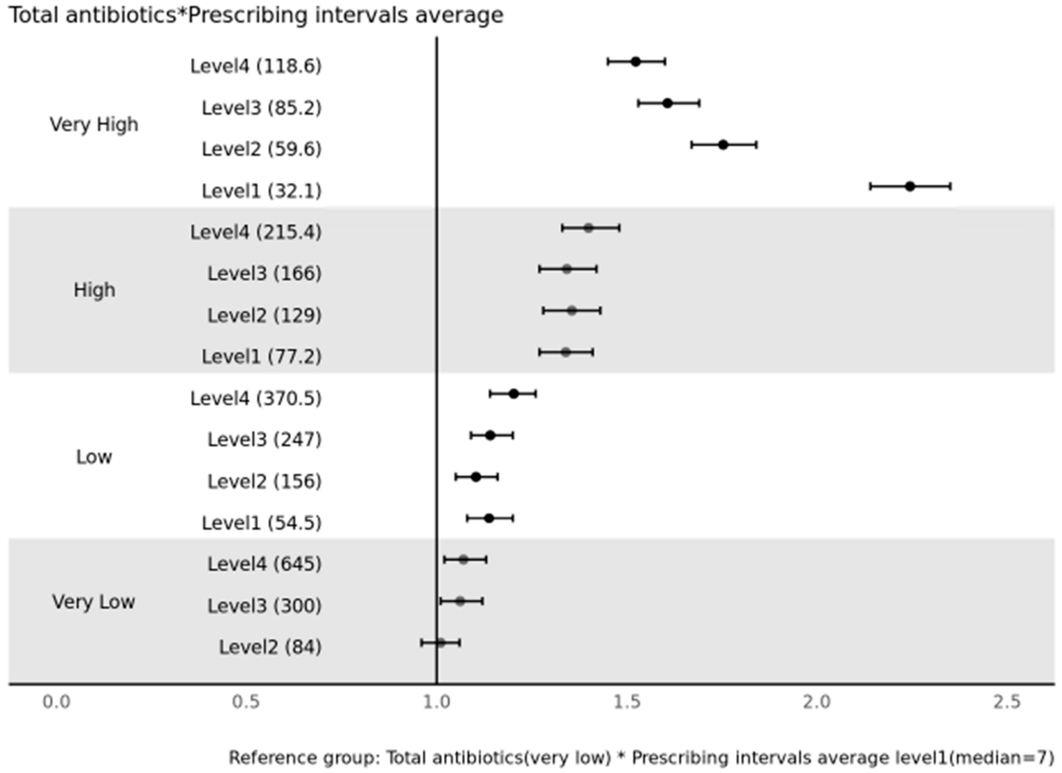

F. Prescribing intervals and deviations stratified by frequency of total antibiotics

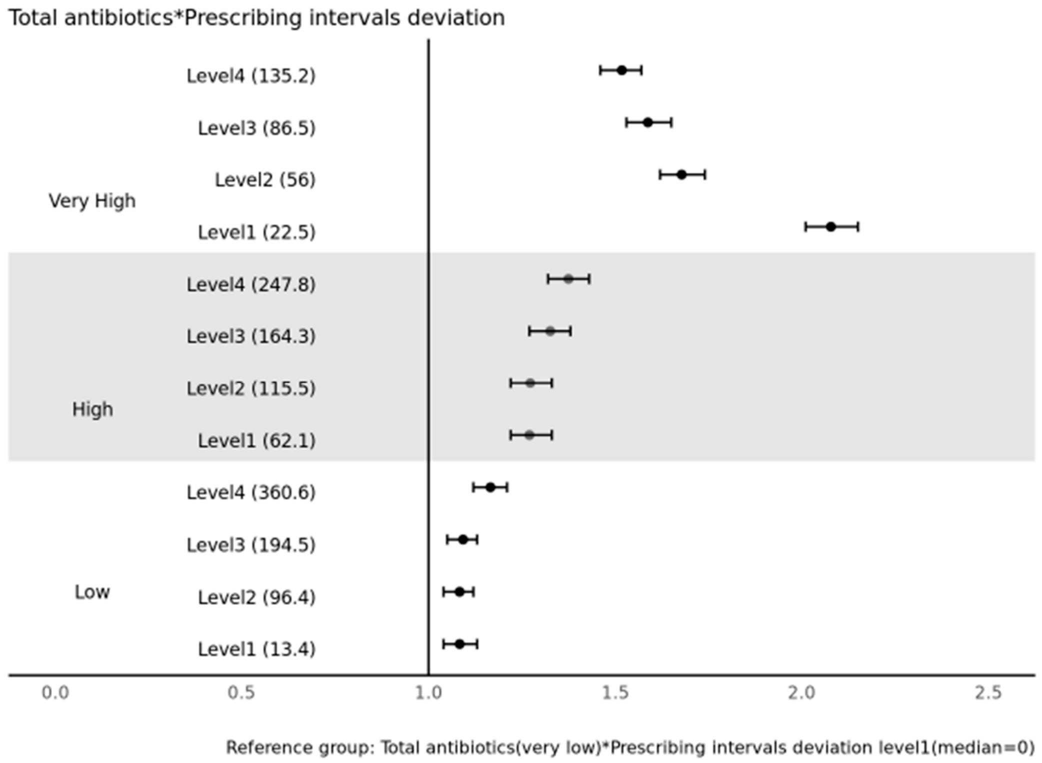

Tables

Supplementary Table S1. Code lists used for variable definition.

1. Code lists are available at https://www.opencodelists.org/
2. Link: https://www.opencodelists.org/codelist/ add **code list version**

Variable type	variable	**Code list version**
Outcome	COVID-19 codes in primary care	opensafely/covid-identification-in-primary-care-probable-covid-clinical-code/24391856<br>opensafely/covid-identification-in-primary-care-probable-covid-positive-test/3d488b8b<br>opensafely/covid-identification-in-primary-care-probable-covid-sequelae/0b29a521
Exposure	All antibiotics	user/BillyZhongUOM/brit_new_dmd/792101bd
Exposure	Broad-spectrum antibiotics	opensafely/co-amoxiclav-cephalosporins-and-quinolones/0d299a50
Exposure	79 types of antibiotics	1. user/yayang/codes_ab_type_amikacincsv/1541da32<br>2. user/yayang/codes_ab_type_amoxicillincsv/7c3266fc<br>3. user/yayang/codes_ab_type_ampicillincsv/6322f3c7<br>4. user/yayang/codes_ab_type_azithromycincsv/4a138092<br>5. user/yayang/codes_ab_type_benzylpenicillincsv/17f49a28<br>6. user/yayang/codes_ab_type_cefaclorcsv/7ee526f2<br>7. user/yayang/codes_ab_type_cefadroxilcsv/65d5b3bd<br>8. user/yayang/codes_ab_type_cefalexincsv/4cc64088<br>9. user/yayang/codes_ab_type_cefiximecsv/688873b3<br>10. user/yayang/codes_ab_type_cefotaximecsv/4f79007e<br>11. user/yayang/codes_ab_type_cefradinecsv/391c4d3f<br>12. user/yayang/codes_ab_type_ceftriaxonecsv/06fd66d5<br>13. user/yayang/codes_ab_type_cefuroximecsv/6dedf39f<br>14. user/yayang/codes_ab_type_chloramphenicolcsv/54de806a<br>15. user/yayang/codes_ab_type_ciprofloxacincsv/22bf9a00<br>16. user/yayang/codes_ab_type_clarithromycincsv/09b026cb<br>17. user/yayang/codes_ab_type_clindamycincsv/70a0b395<br>18. user/yayang/codes_ab_type_co-amoxiclavcsv/57914060<br>19. user/yayang/codes_ab_type_co-fluampicilcsv/3e81cd2b<br>20. user/yayang/codes_ab_type_colistimethatecsv/257259f6<br>21. user/yayang/codes_ab_type_daptomycincsv/41348d21<br>22. user/yayang/codes_ab_type_demeclocyclinecsv/282519ec<br>23. user/yayang/codes_ab_type_doxycyclinecsv/76063381<br>24. user/yayang/codes_ab_type_ertapenemcsv/5cf6c04c<br>25. user/yayang/codes_ab_type_erythromycincsv/43e74d17<br>26. user/yayang/codes_ab_type_fidaxomicincsv/2ad7d9e2<br>27. user/yayang/codes_ab_type_flucloxacillincsv/11c866ad<br>28. user/yayang/codes_ab_type_fosfomycincsv/78b8f377<br>29. user/yayang/codes_ab_type_fusidatecsv/5fa98042<br>30. user/yayang/codes_ab_type_gentamicincsv/469a0d0d<br>31. user/yayang/codes_ab_type_levofloxacincsv/2d8a99d8<br>32. user/yayang/codes_ab_type_linezolidcsv/147b26a3<br>33. user/yayang/codes_ab_type_lymecyclinecsv/7b6bb36d<br>34. user/yayang/codes_ab_type_meropenemcsv/625c4038<br>35. user/yayang/codes_ab_type_methenaminecsv/494ccd03<br>36. user/yayang/codes_ab_type_metronidazolecsv/303d59ce<br>37. user/yayang/codes_ab_type_minocyclinecsv/7e1e7363<br>38. user/yayang/codes_ab_type_moxifloxacincsv/650f002e<br>39. user/yayang/codes_ab_type_neomycincsv/32f019c4<br>40. user/yayang/codes_ab_type_nitazoxanidcsv/00d1335a<br>41. user/yayang/codes_ab_type_nitrofurantoincsv/67c1c024<br>42. user/yayang/codes_ab_type_ofloxacincsv/35a2d9ba<br>43. user/yayang/codes_ab_type_oxytetracyclinecsv/1c936685<br>44. user/yayang/codes_ab_type_phenoxymethylpenicillincsv/0383f350<br>45. user/yayang/codes_ab_type_piperacillincsv/6a74801a<br>46. user/yayang/codes_ab_type_pivmecillinamcsv/51650ce5<br>47. user/yayang/codes_ab_type_rifaximincsv/1f46267b<br>48. user/yayang/codes_ab_type_sulfadiazinecsv/0636b346<br>49. user/yayang/codes_ab_type_teicoplanincsv/08e9733c<br>50. user/yayang/codes_ab_type_telithromycincsv/6fda0006<br>51. user/yayang/codes_ab_type_temocillincsv/2cb088e8<br>52. user/yayang/codes_ab_type_tetracyclinecsv/56ca8cd1<br>53. user/yayang/codes_ab_type_tinidazolecsv/0b9c3332<br>54. user/yayang/codes_ab_type_trimethoprimcsv/597d4cc7<br>55. user/yayang/codes_ab_type_vancomycincsv/406dd992

		not identified:<br>1. user/yayang/codes_ab_type_aztreonamcsv/31040d5d<br>2. user/yayang/codes_ab_type_cefamandolecsv/33b6cd53<br>3. user/yayang/codes_ab_type_cefazolincsv/1aa75a1e<br>4. user/yayang/codes_ab_type_cefepimecsv/0197e6e9<br>5. user/yayang/codes_ab_type_cefoxitincsv/36698d49<br>6. user/yayang/codes_ab_type_cefpiromecsv/1d5a1a14<br>7. user/yayang/codes_ab_type_cefpodoximecsv/6b3b33a9<br>8. user/yayang/codes_ab_type_cefprozilcsv/522bc074<br>9. user/yayang/codes_ab_type_ceftazidimecsv/200cda0a<br>10. user/yayang/codes_ab_type_cilastatincsv/3bcf0d35<br>11. user/yayang/codes_ab_type_dalbavancincsv/0c62e6c1<br>12. user/yayang/codes_ab_type_dalfopristincsv/5a440056<br>13. user/yayang/codes_ab_type_doripenemcsv/0f15a6b7<br>14. user/yayang/codes_ab_type_nalidixic-acidcsv/4bff8cf9<br>15. user/yayang/codes_ab_type_netilmicincsv/19e0a68f<br>16. user/yayang/codes_ab_type_norfloxacincsv/4eb24cef<br>17. user/yayang/codes_ab_type_pristinamycincsv/385599b0<br>18. user/yayang/codes_ab_type_sulfamethoxazolecsv/6d274010<br>19. user/yayang/codes_ab_type_sulfapyridinecsv/5417ccdb<br>20. user/yayang/codes_ab_type_taurolidincsv/3b0859a6<br>21. user/yayang/codes_ab_type_tedizolidcsv/21f8e671<br>22. user/yayang/codes_ab_type_ticarcillincsv/3dbb199c<br>23. user/yayang/codes_ab_type_tigecyclinecsv/24aba667<br>24. user/yayang/codes_ab_type_tobramycincsv/728cbffc
Confounder	Charlson Comobidities	user/yayang/charlson01_cancer/796c49a5<br>user/yayang/charlson02_cvd/605cd670<br>user/yayang/charlson03_copd/474d633b<br>user/yayang/charlson04_heart_failure/2e3df006<br>user/yayang/charlson05_connective_tissue/152e7cd1<br>user/yayang/charlson06_dementia/7c1f099b<br>user/yayang/charlson07_diabetes/630f9666<br>user/yayang/charlson08_diabetes_with_complications/4a002331<br>user/yayang/charlson09_hemiplegia/30f0affc<br>user/yayang/charlson10_hiv/17e13cc7<br>user/yayang/charlson11_metastatic_cancer/7ed1c991<br>user/yayang/charlson12_mild_liver/65c2565c<br>user/yayang/charlson13_mod_severe_liver/4cb2e327<br>user/yayang/charlson14_moderate_several_renaldiseae/33a36ff2<br>user/yayang/charlson15_mi/1a93fcbd<br>user/yayang/charlson16_peptic_ulcer/36562fe8<br>user/yayang/charlson17_peripheral_vascular/68751652
Confounder	Care home residency	primis-covid19-vacc-uptake/longres/v1
Confounder	BMI	primis-covid19-vacc-uptake/bmi_stage/261252c3
Confounder	Smoking	opensafely/smoking-clear/10307fc4<br>opensafely/smoking-unclear/77210c8e
Confounder	Flu vaccination	opensafely/influenza-vaccination/01ec0c67<br>opensafely/influenza-vaccination-clinical-codes-given/443b7295

Supplementary Table S2: Definition of antibiotic-related exposure variables

Antibiotic exposure	Definition	Unit
i. Total antibiotics	total number of antibiotic prescriptions in prior 3 years	Number of prescriptions
ii. Antibiotic types	count of unique type of prior antibiotic prescriptions in prior 3 years	Number of prescriptions
iii. Broad-spectrum antibiotics	count of prior broad-spectrum antibiotic prescriptions in prior 3 years	Number of prescriptions
iv. Time between (first and last antibiotics)	time between the first and the last antibiotic prescription prescriptions in prior 3 years	days
v. Recent antibiotics	time from the last antibiotic prescription prescriptions in prior 3 years until COVID-19 onset	days
vi. Prescribing interval average (mean)	Prescribing intervals were estimated for each individual by collecting the mean number of days between antibiotic prescriptions in the three years prior to index date.	days
vii. Prescribing interval standard deviation (SD)	Standard deviation of number of days between antibiotic prescriptions in the three years prior to index date.	days
*For each patient, the antibiotics prescribed in recent 6 weeks were excluded in this study. The prior 3-year exposure assessment window started from 3 years plus 6 weeks before COVID-19 outcome and completed whole 3-year observation.		

Supplementary Table S3. Characteristics of study cohorts before and after matching

	before matching				matched			
	case		control		case		control	
	n[1]	%	*n*[1]	%	*n*[1]	%	*n*[1]	%
number of patients	69,180	7.8	820,670	92.2	67,515	15.2	375,330	84.8
Inclusion time[2]								
wave 1 of pandemic	11,450	16.6	25,390	3.1	10,905	16.2	57,240	15.3
wave 2 of pandemic	26,125	37.8	232,785	28.4	26,010	38.5	153,290	40.8
wave 3 of pandemic	31,605	45.7	562,500	68.5	30,600	45.3	164,800	43.9
Sex								
female	37,150	53.7	582,745	71.0	36,555	54.1	207,450	55.3
male	32,030	46.3	237,925	29.0	30,960	45.9	167,880	44.7
Mean age (SD)	69.4 (17.2)		45.4 (17.7)		69.2 (17.0)		68.6 (16.8)	
Age group								
18-29	1,755	2.5	174,225	21.2	1,665	2.5	9,115	2.4
30-39	3,255	4.7	168,815	20.6	3,205	4.7	18,350	4.9
40-49	4,805	6.9	157,410	19.2	4,735	7.0	27,040	7.2
50-59	8,395	12.1	142,755	17.4	8,310	12.3	48,065	12.8
60-69	11,200	16.2	89,145	10.9	11,070	16.4	63,885	17.0
70-79	16,735	24.2	53,935	6.6	16,535	24.5	94,630	25.2
80+	23,035	33.3	34,390	4.2	21,995	32.6	114,245	30.4
Practice region								
East	14,875	21.5	183,940	22.4	14,580	21.6	81,215	21.6
East Midlands	13,400	19.4	147,790	18.0	13,020	19.3	70,935	18.9
London	3,960	5.7	39,065	4.8	3,865	5.7	22,245	5.9
North East	4,480	6.5	47,160	5.7	4,435	6.6	25,215	6.7
North West	7,430	10.7	93,845	11.4	7,270	10.8	41,510	11.1
South East	4,195	6.1	43,560	5.3	4,000	5.9	21,595	5.8
South West	5,880	8.5	89,350	10.9	5,690	8.4	30,265	8.1
West Midlands	3,805	5.5	36,920	4.5	3,545	5.3	17,905	4.8
Yorkshire and Humber	11,155	16.1	139,030	16.9	11,110	16.5	64,450	17.2
1. The counts of patients were round to nearest 5 number in line with disclosure controls.								
2. Wave 1: February to August, 2020; Wave 2: September 2020 to April 2021; Wave3: May to December, 2022								

Supplementary Table S4: Antibiotic exposure stratified by outcome

	Overall	Case		Control	
**Variables[1]**	***Median (Q1, Q3)[2]***	***n[3]***	**%**	***n[3]***	**%**
Total antibiotics (count)[4]		9.7 (15.7)		6.9 (10.3)	
Level 1 (lowest)	2 (2,2)	14,145	21.0	105,660	28.2
Level 2	3 (3,4)	17,365	25.7	112,145	29.9
Level 3	6 (5,6)	13,460	19.9	70,555	18.8
Level 4 (highest)	13 (9,21)	22,545	33.4	86,970	23.2
Antibiotic types (count)[5]		2.9 (1.6)		2.6 (1.4)	
Level 1 (lowest)	2 (1,2)	33,390	49.5	215,970	57.5
Level 2	3 (3,3)	15,460	22.9	81,515	21.7
Level 3	4 (4,5)	18,665	27.6	77,850	20.7
Broad-spectrum antibiotics (count)[6]		1.0 (4.5)		0.7 (3.1)	
Level 1 (lowest)	0 (0,0)	46,170	68.4	277,395	73.9
Level 2	1 (1,1)	11,145	16.5	56,910	15.2
Level 3 (highest)	3 (2,5)	10,200	15.1	41,025	10.9
Time between (day)[7]		614.7 (337.9)		542.7 (338.5)	
Level 1 (lowest)	75 (15,163)	13,545	20.1	97,170	25.9
Level 2	423 (343,504)	15,280	22.6	95,565	25.5
Level 3	728 (661, 791)	17,040	25.2	93,870	25.0
Level 4 (highest)	977 (918, 1032)	21,650	32.1	88,725	23.6
Recent antibiotics (day)[8]		275.0 (247.1)		325.9 (264.4)	
Level 1 (lowest)	65 (53,81)	21,770	32.2	89,400	23.8
Level 2	155 (125,190)	17,320	25.7	92,955	24.8
Level 3	334 (282,393)	15,165	22.5	95,850	25.5
Level 4 (highest)	678 (566,825)	13,260	19.6	97,125	25.9
Prescribing intervals average (day)[9]		167.1 (167.1)		183.9 (180.2)	
Level 1 (lowest)	30 (13,47)	18,310	27.1	92,410	24.6
Level 2	93 (78,109)	18,175	26.9	92,580	24.7
Level 3	170 (147,199)	16,370	24.2	94,315	25.1
Level 4 (highest)	363 (287,503)	14,660	21.7	96,030	25.6
Prescribing intervals deviation (day)[9]		101.4 (112.9)		98.6 (116.7)	
Level 1 (lowest)	0 (0,0)	15,160	22.5	112,680	30.0
Level 2	37 (18,53)	17,175	25.4	76,410	20.4
Level 3	103 (85,123)	18,405	27.3	92,305	24.6
Level 4 (highest)	226 (180,309)	16,775	24.8	93,935	25.0

1. continuous variables: mean (SD); and grouped by levels as categorical variables: patient number and percentage
2. values of each level are shown as median and Q1 (25th percentile) and Q3 (75th percentile) number
3. counts of patients rounded to nearest 5 number in line with disclosure controls
4. count of total antibiotic prescriptions grouped by quartile, level 1 is the lowest (1st quartile), level 4 is the highest quartile (4th quartile)
5. count of unique antibiotic type grouped by quartile, level 1 is the lowest (combined 1st and 2nd quartile for same value), level 4 is the highest quartile (4th quartile)
6. count of broad-spectrum antibiotic prescriptions grouped by quartile, level 1 is the lowest (combined 1st and 2nd quartile for same value), level 4 is the highest quartile (4th quartile)
7. time between the first prescription and the last antibiotic prescription, days was estimated and grouped by quartile, level 1 is the lowest (1st quartile), level 4 is the highest quartile (4th quartile)
8. time from the last antibiotic prescription until COVID-19 onset, days was estimated and grouped by quartile, level 1 is the lowest (1st quartile), level 4 is the highest quartile (4th quartile)
9. prescribing interval were estimated by collecting the number of days between each antibiotic prescription by individuals, then work out the mean and standard deviation, values were grouped by quartile, level 1 is the lowest (1st quartile), level 4 is the highest quartile (4th quartile)
